# Effect of Wearable Activity Tracker Social Behaviors on Physical Activity and Exercise Self-Efficacy: Real-World Pilot Study

**DOI:** 10.2196/75133

**Published:** 2026-05-05

**Authors:** Amanda Bireline, Derek J Hevel, Paul G Davis

**Affiliations:** 1 Department of Health & Exercise Science, Mercy University, 555 Broadway, Dobbs Ferry, NY, 10522, United States, 1 914-323-2322; 2 Department of Kinesiology, University of North Carolina at Greensboro, Greensboro, NC, United States

**Keywords:** wearable activity trackers, wearable devices, fitness trackers, physical activity, self-efficacy, health behavior, behavior change, social engagement

## Abstract

**Background:**

Wearable activity trackers are useful tools to track and monitor physical activity (PA), especially considering their use in free-living environments. Users often see moderate improvements in step count, but consistent increases at various intensities of PA are inconclusive. While wearable research is growing, no known studies specifically examine the relationship between how the use of self-selected social features on wearables affects PA and exercise self-efficacy.

**Objective:**

This study aims to compare weekly PA, approximating moderate-to-vigorous intensity, of adults from the New York City metropolitan area assigned to either use or not use social engagement PA features on their device. Exercise self-efficacy was also measured. Additionally, a preliminary examination into the use of 3 different social features was conducted to inform where controlled parameters on feature use may be needed in future work.

**Methods:**

The researchers conducted a real-world pilot study by recruiting wearable users aged 18 years and older in the New York City area to wear their devices in free-living environments. After consent, participants were randomized into 1 of 2 conditions: the condition that involved use of the social engagement PA features or the condition that did not for 8 weeks. Participants submitted objective data from their device and completed a self-efficacy measure at baseline, week 4, and week 8. Those in the intervention group also answered questions about which social feature they used the most throughout the study.

**Results:**

Data from 123 participants were analyzed using mixed methods analysis. Principal findings included no difference between wearable social feature users and nonusers in weekly PA (*P*=.55) or exercise self-efficacy (*P*=.47). There was an overall effect of time across the repeated measures on PA (*P*=.006) with an average increase of 72 (SD 3) minutes. Secondary findings highlight the need to control for the use of only a single social feature to identify more concrete effects. An effect of time was found across the repeated measures (*P*=.01) in the intervention group, showing an increase of 49 to 126 minutes of PA, depending on the feature used most. The mixed methods analysis also found that exercise self-efficacy did not significantly change based on which social feature was used most (*P*=.24).

**Conclusions:**

Consistent with other literature, this pilot study demonstrates that using wearables can lead to increases in PA and that sharing one’s PA data with others may amplify the effect. However, the novelty of this study is that although carefully implied, specific social features on a wearable may have a greater effect than others. This study identified the need for further investigation into which features may be more effective. With the increased prevalence of device ownership, knowing if certain social features lead to greater increases in PA may help those encouraging PA behavior change.

## Introduction

### Background

There has been a vast decrease in adequate physical activity (PA) participation over the past 3 decades [1-6]. Physical inactivity is a term used to describe a state in which individuals do not meet health-related PA recommendations [7]. The current weekly aerobic and muscle-strengthening recommendations include 150 minutes or more of moderate intensity or 75 minutes or more of vigorous intensity aerobic activity and 2 nonconsecutive days or more of muscle-strengthening activity [8]. While inactivity is a worldwide issue, low levels of activity are especially concerning in the United States. The most recent report from the Centers for Disease Control and Prevention found that only 26% of US adults (≥18 y) met the weekly aerobic and muscle-strengthening recommendations. Adequate participation in PA, generally in the moderate-to-vigorous-intensity range, is known to result in numerous health benefits, including lower all-cause and cardiovascular disease mortality and reduction in the prevalence of other chronic diseases [9]. Many of the health challenges related to inactive lifestyles can be reduced or eliminated with regular PA. Because of this, PA interventions that address suboptimal activity levels have been implemented to help increase activity and reduce the risk of developing or progressing health conditions. Addressing this issue may be particularly important for the young adult population to help reduce the risk of developing health conditions later in life.

Recently, the rapid growth in wearable trackers that incorporate behavior change techniques (eg, goal setting, self-regulation, social support) has led to their use in PA interventions. Wearable activity trackers, typically worn as smartwatches, are everyday devices that help users monitor health-related metrics (eg, sleep patterns) and PA data (eg, step count) in real time. Real-world insights can be gleaned from wearable tracker users’ activity data, as these devices allow users to track their metrics independently in free-living environments and are more cost-effective than laboratory assessments [10,11]. This is important as it allows for a more realistic assessment of wearable tracker implementation in day-to-day life. An uptick in PA social sharing from these devices to platforms, such as Instagram and Facebook, has also been noted [5]. Research on the use of wearables to improve PA has shown increases in daily step count and exercise intention, and these approaches are being implemented in clinical interventions [1-4]. Although wearable features (eg, self-regulation, goal setting) offer multicomponent interventions that may potentiate behavior change [12,13], studies using wearable trackers have been somewhat inconclusive on other more specific PA metrics, such as light versus moderate-to-vigorous physical activity (MVPA) [1-4,14,15]. While many studies have been conducted exploring how social environments impact PA through app- and web-based interventions, to date, no known studies have specifically looked at the use of social elements on a smartwatch and social engagement through the wearable features themselves.

### Theoretical Framework

Theories and models used to address health-related behavior change (eg, social cognitive theory [SCT], health belief model) often focus on the social foundations of behavior choice. Particularly, social support has been found to help reduce barriers to PA and increase self-efficacy [16,17]. The social presence and observation of others’ behaviors may cause a person to act or adjust their behaviors based on the perceived positive outcomes [18]. While the positive effect of social elements on PA is well documented [19-21], research is somewhat limited to face-to-face contexts where more active participation and higher social engagement may occur compared to more passive or lower engagement interventions or does not delineate which social element may be more influential. Additionally, studies have been conducted on app or web-based interventions that use social engagement and PA, but these types of delivery may be less accessible compared to a wearable that may be worn on a person’s wrist for a majority of wake time. Therefore, it would be helpful to know if, along with wearable tracker behavior change techniques, engaging with wearable social features related to exercise will help increase PA, particularly at intensities that optimize positive health changes. With wearable device ownership rising, it is essential to learn how social features affect PA and how they might positively influence exercise self-efficacy, a mediator of activity engagement [22].

SCT was used to frame this study, as it is the most widely used behavior model in PA interventions and shows the greatest amount of evidence for predicting, explaining, and intervening in activity engagement [23,24]. SCT postulates that learning occurs in social contexts with both dynamic and reciprocal interactions among the person, the environment, and the behavior [25]. This theory consists of constructs (outcome expectations, self-efficacy, sociostructural factors [ie, facilitators and barriers], and goals) that impact behavior and seek to understand behavioral regulation through control and reinforcement [26].

### Research Objectives

The principal purpose of this pilot study was to examine whether using wearable trackers (ie, smartwatch) with social features had a greater influence on PA at approximately the moderate-to-vigorous level and exercise self-efficacy compared to not using them in metropolitan-dwelling adults. This study also evaluated how changes in exercise self-efficacy affect changes in PA among participants. Secondary outcomes were also assessed to determine if there was a specific feature used more than the others (social comparison, competition, or social support) that had a greater effect on these measures to inform where future controls on feature use may need to be used.

## Methods

### Study Design and Setting

This pilot study was conducted to assess the potential of wearable tracker social features to increase MVPA in a free-living environment. It used a pretest, mid-test, and posttest design to evaluate participants’ weekly exercise minutes gathered from their wearable devices (Apple Watch) and exercise self-efficacy scores gathered from digital surveys over an 8-week period. All study procedures were carried out in the New York City metropolitan area. SCT guided this behavioral assessment study based on its constructs (eg, self-efficacy, facilitators, and barriers) and their relevance to health-related behavior change.

### Eligibility Criteria

Adults (≥18 y) living in the New York City metropolitan area who were Apple Watch users were recruited to participate in this study. Only Apple Watch users who reported not using the device’s exercise social features were allowed to participate. This specific wearable was chosen, as it is currently the most used wearable tracker [27]. Exclusion criteria included individuals who were pregnant, suspected that they might be pregnant, or who had been told by their physician to not currently participate in higher levels of PA for any other reason. The participants were screened for eligibility by responding to survey questions about the aforementioned criteria.

### Recruitment

To determine the appropriate number of participants needed to find significance in changes in weekly exercise minutes, a priori power analysis using the G*Power software (version 3.1.9.7) was used, and it revealed that 102 participants (51 in each group) were needed to detect an effect size of Cohen *d* of 0.8 with 80% power (*α*=.05). The recruitment of participants was conducted via flyers posted by one of the researchers in public facilities, such as recreation centers and community boards, across the campuses of Mercy University and by word of mouth in the New York City metropolitan area.

### Ethical Considerations

This study was approved by the institutional review board at the University of North Carolina at Greensboro (UNCG-IRB-FY23-586). Per ethical guidelines, informed consent was obtained from all participants prior to data collection. The consent included benefits and risks associated with participating in the study. The data were deidentified and coded immediately upon study enrollment, and each participant was assigned a subject ID number to ensure all data were kept private and confidential. Data collection occurred from July to December 2023. No compensation was given to participants for participating in this study.

### PA Measures

PA data collection included exercise minutes from the participants’ wearable trackers. Although not listed specifically as MVPA, the Apple Watch automatically measures “exercise minutes” during all wear time, which are specific to the user based on biometric data and algorithms. Exercise minutes are recorded for “every full minute of movement equal to or exceeding the intensity of a brisk walk” [28]. More precisely, the algorithms use the device wearer’s age, height, weight, sex, heart rate, motion data (via accelerometry and Global Positioning System), and cardiorespiratory fitness level (ie, estimated maximal oxygen consumption) to determine what is considered brisk walking intensity [29]. Previous research has validated the use of the Apple Watch to comparably measure MVPA [30-32]. As such, we operationalized “exercise minutes” as MVPA. To track exercise minutes, participants were directed to upload a screenshot of the current week’s data into the surveys at prestudy, midstudy, and poststudy (ie, 0, 4, and 8 weeks).

### Self-Efficacy Measures

To measure exercise self-efficacy, the Resnick & Jenkins 9-item Self-Efficacy for Exercise Scale (*α*=.92) was used [33]. This scale sums the responses from the 11-point Likert scale (0=not confident to 10=very confident) to provide an overall exercise self-efficacy score ranging from 0 to 90. This scale was completed and submitted at weeks 0, 4, and 8.

### Other Measures

Additional survey questions were completed at baseline to gather participant demographics, current behaviors related to the monitoring of PA on the wearable tracker, daily wear time, and PA habits. In addition, at week 8 (end of data collection), participants in the social feature user condition were asked to report which of the 3 features they used the most during the study and which feature they thought contributed to changes in their MVPA and exercise self-efficacy. All survey measures were created in Qualtrics so that a link could be easily distributed to study participants and can be found in Multimedia Appendix 1.

### Behavioral Assignment

#### Overview

This pilot study used a behavioral encouragement design to randomly assign participants to 1 of 2 groups: one that encouraged users to engage with others about their PA with social features on the wearable tracker and another that asked users to continue using the Apple Watch as normal, not using the social features. This study design allowed for the examination of individual differences across the repeated measures and treatment effects between the conditions and is most appropriate for real-world studies, as it is accepted in these environments that individuals are unlikely to show complete adherence to the intervention [34]. It also follows the intention-to-treat analysis principles, which include all data regardless of protocol violations and loss to follow-up [35]. Additionally, this pilot study used this format due to the selected wearable tracker and its design of the social feature tool. The Apple Watch allows for full control over which social features device owners engage with; once fitness sharing is activated, all social features are enabled for use without the ability to deactivate any of them.

Upon condition assignment, participants in the social feature condition were given instructions on how to connect with other users directly from their watches or mobile devices, provide customized or prescripted feedback on workouts and activity goals, initiate and engage in weekly competitions, and view others’ activity data to compare to their own in real time (Multimedia Appendix 2). Participants in the social feature condition were also encouraged to engage with all 3 social features for the entire 8 weeks. Individuals who were in the condition that did not involve the use of the social features on their wearable were encouraged to continue with their regular PA habits.

#### Functional Definitions

Fitness sharing on the wearable activity tracker includes the disclosure of activity data with other selected device users. The following definitions specific to the fitness sharing features were provided to participants for accurate responses in the poststudy survey:

- Social comparison: the evaluation and comparison of one’s personal activity data to the activity data of other users on the device. Users can view or compare connections to daily or weekly activity data in real time both on the wearable activity tracker and the associated phone app [36,37].
- Competition: active engagement in the device’s competition feature. Connected users can initiate a repeatable 1-week competition through the device; each party earns 1 point for every full minute of PA at or above the intensity of a brisk walk [37]. Live feedback on competition rankings is provided directly through the device.
- Social support: active engagement in comments and/or “likes” of social connections, workout completion, and earned rewards. When connected with other device users, notifications display on the watch about the connection’s activity progress and earned rewards (eg, completed workout, new personal records), allowing for personalized or automated comments to be made in return [37].

### Study Procedures

After expressing interest in the study, potential participants were screened for eligibility, and consent was obtained. Eligible participants were then randomly assigned (using a random number generator) to the group that was encouraged to use the PA social features on their wearable tracker (ie, “intervention group”) or to the group that was asked not to use the social features about activity on their device (ie, “control group”). Each participant was assigned a subject ID number, and all data were deidentified. The intervention group assignment was not visible to the researcher after initial communication with participants until the final data collection point and submission of the post-study survey. Each participant was sent the prestudy survey at baseline and which group they were assigned to. Those in the intervention group also received instructions to connect and engage with others on their device. At week 4, all participants were sent the midstudy survey to evaluate their exercise self-efficacy and gather exercise minutes. Finally, at the completion of week 8, all participants were sent the poststudy surveys. The intervention group survey included additional questions about the use of the social features throughout the 8 weeks on the participants’ PA and exercise self-efficacy. This study was conducted in accordance with the CONSORT (Consolidated Standards of Reporting Trials) checklist (Checklist 1).

### Statistical Analysis

Primary comparisons were made using mixed model analyses to determine if changes in exercise minutes or self-efficacy differed between social feature users and nonusers. Pearson coefficient correlations were computed to assess relationships between changes in exercise self-efficacy and changes in exercise minutes at various time points in the study. Secondary analyses of the same variables, also using mixed models, were also completed within the social feature users based on which social feature (social comparison, competition, or social support) they reported using most often throughout the study (ie, “feature use grouping”). All participant data were used, regardless of missing values due to not completion of data collection at all 3 time points (5/123, 4%) and computed in SPSS (version 29; IBM Corp). Statistical significance was set at *P*<.05 with a 95% CI for all analyses.

## Results

### Overview and Demographics

**Figure 1.**
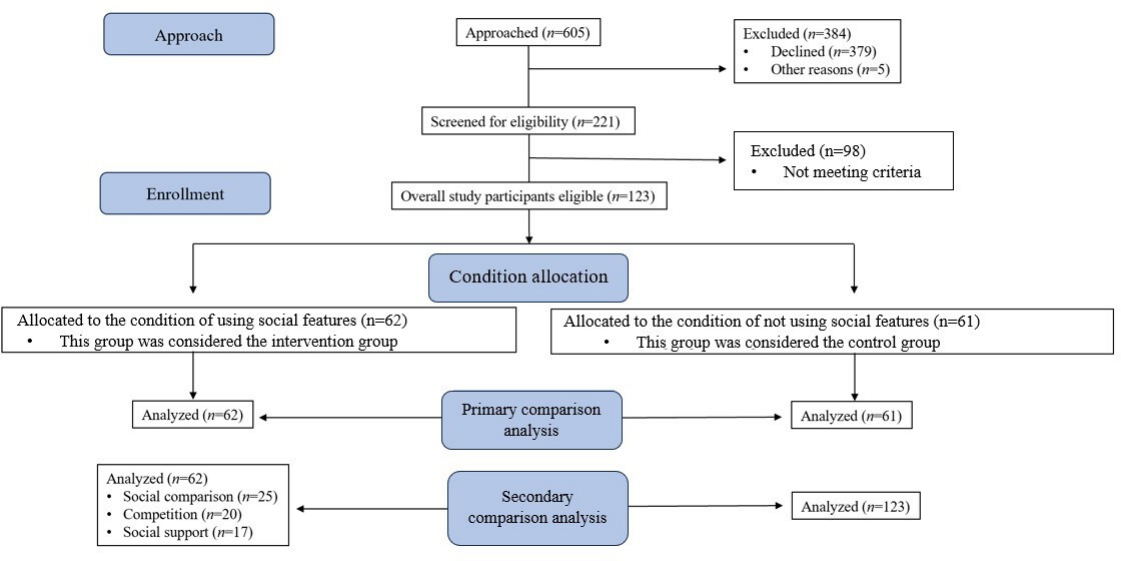
CONSORT (Consolidated Standards of Reporting Trials) flow diagram showing participant recruitment, eligibility screening, enrollment, condition assignment, and analyses for this pilot study.

Figure 1 shows the CONSORT flow of participants starting with the initial recruitment of 605 individuals. After learning more study details, 379 individuals chose not to move forward with participation, and 5 were excluded due to issues related to engagement in PA at least at moderate intensities. Eligibility screening and the exclusion of participants due to pregnancy or current engagement with social features on the wearable left 123 participants to be randomly allocated to the social feature user condition “intervention” (n=62) or the nonuser condition “control” (n=61). These groups were used for primary comparisons. For secondary comparisons, the intervention group was further analyzed based on which social feature they reported using most throughout the study. Table 1 provides a description of enrolled participants’ demographic characteristics, with a mean age of 35 (SD 15; range 18‐71 y), including 85 out of 123 (69%) being female and 107 out of 123 (87%) being city dwellers. In total, 16 out of 123 (13%) participants who lived outside the New York City metropolitan area (5 boroughs) lived in zip codes adjacent to the city.

**Table 1.** Participant demographics (n=123).

Demographic characteristic	Participants, n (%)
Age	
18‐24	40 (32.5)
25‐34	30 (24.4)
35‐44	22 (17.9)
45‐54	11 (9)
55‐64	17 (13.8)
≥65	3 (2.4)
Gender	
Male	37 (30.1)
Female	85 (69.1)
Nonbinary or third gender	1 (0.8)

### Weekly Exercise Minutes of Intervention Versus Control

Among all study participants, repeated measures revealed a significant time effect on weekly exercise minutes with an increase of 72 (SD 3) minutes from the prestudy to poststudy period (*P*=.006; Table 2). There was an effect of time for those in the intervention group (*P*=.002) but not in the control group (*P*=.23). No group interaction existed (*P*=.55).

**Table 2.** Weekly exercise minutes based on intervention (feature users) versus control (nonfeature users).

Research participants	Participants (n=123), n (%)	Baseline weekly exercise minutes, mean (SD)	Week 4 weekly exercise minutes, mean (SD)	Week 8 weekly exercise minutes, mean (SD)
Overall sample	123 (100)	271 (177)	297 (177)	343 (180)^a^^,b^
Randomized condition				
Intervention—social feature users	62 (50.4)	276 (158)	324 (182)	369 (171)^c^
Control—nonfeature users	61 (49.6)	266 (196)	269 (169)	318 (187)

a Different from week 4 (*P*=.03).

b Different from baseline (*P*=.002).

c Different from baseline (*P*=.002).

### Exercise Self-Efficacy of Intervention Versus Control

There was no significant effect of time across the repeated measures on exercise self-efficacy for all participants (*P*=.35) or an interaction between the intervention and control groups (*P*=.47; Table 3).

**Table 3.** Exercise self-efficacy scores based on intervention (feature users) versus control (nonusers).

Research participants	Participants (n=123), n (%)	Baseline exercise self-efficacy score, mean (SD)	Week 4 exercise self-efficacy score, mean (SD)	Week 8 exercise self-efficacy score, mean (SD)
Overall sample	123 (100)	52 (20)	49 (20)	52 (19)
Randomized condition				
Intervention—social feature users	62 (50.4)	55 (19)	49 (20)	53 (19)
Control—nonfeature users	61 (49.6)	49 (20)	49 (19)	51 (20)

### Relationship Between Exercise Minutes and Exercise Self-Efficacy

A Pearson correlation determined a weak positive relationship between the change in exercise self-efficacy and the change in weekly PA from baseline to week 8 for all study participants. To ascertain if higher or lower baseline exercise self-efficacy scores affected changes in exercise minutes across the 8 weeks, another correlation was computed with no relationship being found. A final correlation was calculated to determine if changes in exercise minutes affected self-efficacy scores at the end of the study with no relationship being found (Table 4).

**Table 4.** Relationship of change in exercise minutes (baseline to week 8) to exercise self-efficacy (Pearson *r* and 2-tailed *P* value)^a^.

Variable	Baseline to week 8 exercise minutes
Baseline to week 8 exercise self-efficacy	
*r*	0.21
*P* value	.01
Baseline exercise self-efficacy	
*r*	−0.11
*P* value	.22
Week 8 exercise self-efficacy	
*r*	0.08
*P* value	.33

a Significance of the correlation was set at the .05 level (2-tailed).

### Weekly Exercise Minutes of Social Feature Use Grouping

As stated above, repeated measures analysis revealed a time effect on weekly exercise minutes (+92, SD 42 at week 8 compared to baseline; *P*=.01) within the intervention participants (n=62). Secondary analyses were performed after stratifying these participants based on which feature they reported using the most during the study. Although mixed methods analysis did not indicate that the major use of any social feature (ie, social comparison, competition, or social support) had significantly greater effect than another across the repeated measures (*P*=.13), the overall increase in weekly exercise minutes was driven mostly by those who reported using the social comparison feature the most (+126, SD 129; *P*=.006), while those who reported using the competition (+88, SD 57), and social support (+40, SD 43) features the most contributed less to the overall increase (Table 5).

**Table 5.** Weekly physical activity minutes based on feature use grouping.

	Social feature users (n=62), n (%)	Baseline weekly exercise minutes, mean (SD)	Week 4 weekly exercise minutes, mean (SD)	Week 8 weekly exercise minutes, mean (SD)
Overall social feature users	62 (100)	276 (158)	324 (182)	369 (171)^a^
Top reported social feature use				
Social comparison	25 (40.3)	269 (154)	364 (184)	395 (156)^b^
Competition	20 (32.3)	298 (151)	314 (181)	386 (208)
Social support	17 (27.4)	261 (178)	276 (176)	310 (135)

a Different from baseline (*P*=.006).

b Different from baseline (*P*=.004).

### Exercise Self-Efficacy of Feature Use Grouping

When grouping together all social feature users (n=62), mixed methods analysis resulted in no effect of time (*P*=.24) across the repeated measures on exercise self-efficacy or an interaction (*P*=.81) based on which social feature (ie, social comparison, competition, or social support) the users reported using most during the 8 weeks. Descriptive statistics indicated a decrease in exercise self-efficacy from baseline to week 4 and an increase from week 4 to week 8 among participants grouped by feature use, though no groups reached baseline values (Table 6).

**Table 6.** Exercise self-efficacy scores based on feature use grouping.

	Social feature users (n=62), n (%)	Baseline exercise self-efficacy score, mean (SD)	Week 4 exercise self-efficacy score, mean (SD)	Week 8 exercise self-efficacy score, mean (SD)
Overall social feature users	62 (100)	55 (19)	49 (20)	53 (19)
Top reported social feature use				
Social comparison	25 (40.3)	57 (16)	46 (21)	54 (19)
Competition	20 (32.3)	49 (21)	46 (19)	48 (20)
Social support	17 (27.4)	61 (18)	56 (20)	57 (17)

## Discussion

### Principal Findings

This pilot study set out to explore whether using social features specifically on a wearable tracker (Apple Watch) would have an effect on weekly PA and exercise self-efficacy. We found that weekly PA, approximating moderate-to-vigorous intensity, was significantly increased from baseline to week 8. While all participants showed a significant change in their activity levels across the study (*P*=.006), the change was driven by the intervention group that was assigned to engage with their social features (*P*=.008). The intervention group saw an average increase of 93 (SD 13) minutes of activity per week compared to the control group that showed an average increase of 52 (SD 9) minutes per week.

Conscious monitoring of activity promotes self-regulation, which may have led to the changes still seen in the control group. Being conscious of activity habits through real-time monitoring helps apply the foundations of self-regulation (ie, activate, monitor, and adapt behavior in response to cues and feedback) [38]. This finding aligns with other literature that suggests self-monitoring on wearables may facilitate increased PA [39]. One study looking at how technology-based goal setting affects PA found that significant increases occurred over a 3-month intervention, and, notably, these increases remained 3 months after the intervention was complete [40]. Goal setting on wearables, in conjunction with the use of other features, parallels techniques used in evidence-based clinical interventions [41], and it has been suggested that their use has an equal effect on individuals of all ages and in clinical and nonclinical populations [2]. It is important to note that while this study did not specifically guide participants to set activity goals on their devices, each participant already had predetermined daily goals set. During device setup, the Apple Watch prompts users to set their daily “rings” (ie, calories, brisk activity, and stand time) and provides digital reminders and completion awards. These goals can be adjusted at any time by the user. Thus, self-regulation may have already been occurring to some degree prior to the study.

Given the increasingly high prevalence of wearable tracker ownership, with an anticipated increase of $220.8 billion by 2032 [42], it is important to know that self-regulation consistently has been found to show positive PA behavior change. The results from this study are aligned with previous studies that reported consistent increases in daily step count and MVPA when wearable activity trackers were used as a primary component of interventions and suggest that wearables are effective at increasing conscious PA behaviors [1,3]. Congruently, a key finding of this study was an average increase of 72 (SD 3) minutes of weekly PA (72/150 min, 48% greater than the World Health Organization’s minimum public health recommendations), even though the average participant was comfortably meeting the PA recommendations at baseline (271 min/wk). Importantly, this study’s sample consisted of urban dwellers who likely walked more than the average person for the purpose of transportation. It is notable that conscious use of a wearable activity tracker increased exercise minutes by such a considerable amount. While external validity may not be as strong due to high baseline activity levels, these results speak to the impact that wearable trackers may have on individual weekly PA, likely due to self-regulation on the device.

For the intervention group that showed a more significant change in their weekly PA, more in-depth exploration into how social environments on digital devices may affect behavior is warranted and worth discussion. Duncan et al [43] examined 3 common wearable trackers and reported an average of 18 PA-related behavior change techniques implemented among devices and highlighted that some of these techniques involved social interaction (eg, “provide information about others’ approval,” “provide normative information about others’ behavior,” “prompt identification as role model or position advocate”). Accordingly, support from other device users has been found to have a significant increase in step count [17], although this is not always the case [44]. Regardless, it is generally understood that social components often result in greater effects. Peterson et al [45] found that app-specific communities result in an increase in PA when using the social components. A 2019 systematic review determining if app-based PA interventions that included social elements increased activity levels more than ones without social integration discovered similar findings [46]. While this study is the first of its kind to look specifically at social engagement on a smartwatch and PA, the results appear consistent that there is an estimable treatment effect with that of app- and web-based interventions; however, more work should be done to validate these findings

With respect to exercise self-efficacy, this study found that there was no significant change in self-efficacy scores regardless of using social features on a wearable tracker or not. There was also not an effect of time across the repeated measures for the intervention or control group. While self-efficacy and PA are often related, there may be explainable reasons why the results did not show a change over the study’s duration. Prior studies have found that, after controlling for baseline behavior, self-efficacy was a significant predictor of PA and suggested that it is a modifiable construct of behavior change [47,48]. Numerous activity interventions focused on increasing self-efficacy have been implemented in face-to-face environments and on app- and web-based platforms across all ages and physical capabilities, with some resulting in increased PA [49-52]. Many researchers express that self-efficacy may be a mediator to increase PA, but likely in conjunction with other elements such as intention, removal of barriers, or motivation. Self-efficacy in this study, regardless of whether or not social features were used, was not higher after 8 weeks of wearable use versus baseline. In fact, self-efficacy decreased from baseline to week 4, likely due to inflated baseline scores, rebounded, and even improved by week 8 for the control group. The intervention group that was using social features on their wearable tracker during the study found the same result, a drop in self-efficacy from baseline to week 4 but did not see a rebound to baseline values at week 8. The identity theory explains that individuals often inflate responses as they want to appear consistent with their desired self [53]. Changes in self-efficacy can be highly contextual and transient [54]. Social feature users may have potentially relied too much on interaction with others rather than self-reliance toward PA. While literature suggests using techniques such as vicarious experiences and verbal persuasion may enhance self-efficacy [55,56], this study may not have been long enough or provided sufficient background knowledge on the importance of meeting PA guidelines to effect change. While the use of wearable activity trackers may provide information that can enhance self-efficacy (eg, performance accomplishments), these devices may not address task difficulty or provide an evaluation of external circumstances to a significant degree [57]. Additionally, because participants were already device users, the motivation or novelty of wearing the smartwatch may have already worn off or plateaued.

Changes in exercise self-efficacy and PA were also evaluated. The Pearson correlation coefficient determined a weak but positive relationship between changes in each of these measures, aligning with prior research on the relationship between the two [47,48]. We also wanted to evaluate whether high or low baseline and week 8 (end of study) self-efficacy had an effect on weekly PA and found that no relationship existed.

### Secondary Findings

Given the design of the selected wearable tracker used in this study and its capability to engage socially in 3 different ways (ie, social comparison, competition, social support), preliminary data were gathered from responses from participants in the intervention group to inform necessary adjustments to study protocols in future work. As such, participants in the intervention group were asked to engage with all 3 social features during the 8 weeks and report on which feature they used the most (ie, “feature use grouping”). PA and exercise self-efficacy were evaluated based on those responses.

These preliminary findings suggest there was an effect of time on PA across the repeated measures with an average increase of 93 (SD 42) minutes regardless of which feature participants used most. Regardless of which social feature was reported as being used the most, there was no significant change in exercise self-efficacy. When looking at feature group use, only those who reported using social comparison the most showed an increase. This considerable increase may have resulted, in part, from exposure to the PA behaviors of others and adjusting due to social influence. Bandura’s SCT highlights that learning occurs in social contexts, and by exposure to the behavior of others, one may adjust based on the associated outcomes [25]. An additional construct under SCT is what Young et al [26] term “sociostructural factors” or facilitators and barriers. The connections to recent and live activity of others may be a facilitator to increase PA in some users.

We controlled for social feature use to the degree to which the activity tracker used in this study allows. However, although the participants in our study reported which social feature they used the most, this did not restrict them from using the other 2 features. Thus, the results from these secondary findings should be interpreted with caution. Also, although the group using the Social Comparison feature the most showed the greatest increase in exercise minutes, there were varied responses within each group, and we cannot rule out that some participants might have benefited more from using the competition or social support features. Social feature preference may be related to factors such as device design, level of required engagement, or individual preference for social feature reliance to spur on activity. One person may seek social support for motivation and acceptance, while another already has higher levels of social support and may prefer engaging in a competition or quietly comparing themselves to others. This aligns with Alley et al [46], who found that individuals with already high social support did not see an increase in MVPA when a social support intervention was implemented. In addition to social feature preference, technology acceptance could be a contributing factor to engagement with specific social features. According to the technology adoption model, the acceptance and use of the features may depend on how the user interprets their usefulness and ease of use [58-60], along with other factors such as age, presumption of data accuracy, attitude, and motivation [60]. If a user is not inherently competitive or if they are consistently unsuccessful in competitions, their likelihood of adoption will likely decrease. On the contrary, if the opinions of others are important to them, they may then place more value on the usefulness of the social support feature, further increasing their perception of ease of use. These results are important to inform future research needs, particularly around controlling for single social feature use.

### Strengths and Limitations

This pilot study does have limitations. The inclusion criterion of being an Apple Watch owner reduces the potential participant pool and external validity, as it singles out one type of wearable activity tracker. The cost of these devices also creates an economic bias [61]. In addition, with participants already being technologically immersed (eg, smartwatch owners), it may be challenging to generalize the results to populations who may be less comfortable with technology. Furthermore, the length of the data collection may not accurately represent PA behaviors over the long term, and device compliance and accurate reporting of social feature use may impact precise measures. Although 106 out of 123 of our participants (86%) averaged wearing their device more than 7 hours per day, providing specific parameters may be important in future work. This study was also conducted in a large metropolitan area where many rely on commuting via foot or public transit rather than using automobiles, which likely explains the high baseline PA reported. It is also important to note that the smartwatch used in this study does not explicitly equate “exercise minutes” to MVPA and computes exercise minutes through the device’s algorithmic calculations rather than using traditionally accepted standards (ie,≥3 METs), although the device’s support material (ie, “equal to or exceeding the intensity of a brisk walk”) suggests that the two are closely aligned.

Nevertheless, knowing how specific features on wearable trackers affect PA and exercise self-efficacy, even in short-term bouts, may allow for implementation in appropriate contexts and bring about awareness of activity habits. Although previous studies have reported how using social features in app- or web-based interventions affect PA, the accessibility of a smartwatch worn during wake hours and viewable during times when accessing a smartphone app may not be as feasible adds contemporary information to the literature. Numerical data were also collected in free-living environments, allowing for more practical implementation. The Apple Watch, along with other wearables (eg, Fitbit, Garmin), already has these built-in social and behavior change features, so observing the potential influence that some features may have over others can allow for immediate implementation rather than necessitating a new technological design. With these devices being a part of many individuals’ everyday attire, they can easily be used to promote PA engagement in several contexts.

### Future Research Needs

The results from this study suggest that using PA social features on a smartwatch may increase weekly PA, but the type of interaction among users might be important as well. This pilot study was conducted to determine if the results were consistent with previous results from app- and web-based interventions on PA and highlighted the need to adjust the current protocol and conduct further studies on individual social components. Additionally, while PA increased substantially, the majority of our participants were young and female. Thus, future research should implement more targeted recruitment of other genders and age groups to identify if consistent findings exist to allow for more generalizability. Additionally, studies should intentionally recruit less active individuals in geographical areas that are minimally dependent on foot traffic to allow for greater generalizability and relevance to public health recommendations. We are currently designing a study that uses these pilot methods but will intentionally recruit a more balanced sample (ie, gender and age distribution) and involve the use of just 1 specific social feature at a time.

### Conclusions

As the world becomes more technologically immersed, understanding how wearables can be used to promote healthy habits may provide long-term benefits to users. With these commercially available devices already integrated into the daily lives of 563 million individuals [62], the wide-scale application of effective PA promotion tools is warranted and plausible. While this study supports the current knowledge base that wearable tracker wearers engage in more weekly PA when consciously monitoring it, it also adds new insights as to how engagement specifically on a smartwatch, as opposed to web- or app-based programming on a smartphone, impacts activity behaviors. Additionally, it highlights the need for future work on evaluating the influence of specific social features on PA participation. Thus, device use to set and monitor activity goals can be encouraged where increased PA is a focus, and it should be noted that when wearable users share their PA data and observe those of others (ie, social comparison), this may lead to increased weekly PA. Professionals can help connect wearable activity tracker users and guide them toward features that would have the greatest effect.

## Supplementary material

10.2196/75133 Multimedia Appendix 1 Prestudy, midstudy, and poststudy surveys.

10.2196/75133 Multimedia Appendix 2 Social feature user participant instructions.

10.2196/75133 Checklist 1 CONSORT checklist for pilot or feasibility study.

## Abbreviations

CONSORT: Consolidated Standards of Reporting Trials
MVPA: moderate-to-vigorous physical activity
PA: physical activity
SCT: social cognitive theory
