# Supplementary material for: Effect of Wearable Activity Tracker Social Behaviors on Physical Activity and Exercise Self-Efficacy: Real-World Pilot Study
Source: JMIR Form Res. 2026 May 5;10:e75133. doi: 10.2196/75133 (PMC13143198; doi:10.2196/75133)
Supplement: Multimedia Appendix 1 [file formative-v10-e75133-s001.pdf]

Supplementary file: Survey Instruments

Pre-study Survey - Completed at baseline

How much time per day do you spend wearing your Apple Watch? If you don’t know the exact time, please estimate as closely as possible.

- O 1-4hrs.
- O 4-7hrs.
- O 7-10hrs.
- O More than 10hrs.

The Apple Watch provides live metrics on your activity such as total daily exercise minutes, stand time, and movement calories provided on the device or fitness app. These are commonly known as the blue, green, and red “activity rings”.

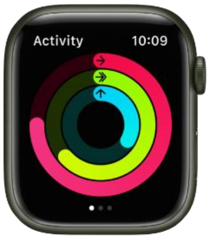

Do you currently monitor your activity rings on your device?

- O Yes
- O No

Do you currently share your activity data with other Apple Watch users?

- O Yes
- O No
- O I am not sure

The Apple Watch has built in social features that you can engage with regarding your activity data. Some of these social features include competing against other connections, "liking" or commenting on your connection's activity progress or comparing your personal activity to others you are connected with on your activity dashboard.

In the last 4-weeks, have you been actively engaging on the built in social features on your Apple Watch with those you are connected with?

- O Yes
- O No

This set of nine questions will be evaluating your confidence level regarding engagement in physical activity. Please select the response that best answers the question prompt right now.

0= Not confident
10= Very confident

	0 = Not confident	1	2	3	4	5	6	7	8	9	10 = Very confident
The weather was bothering you	○	○	○	○	○	○	○	○	○	○	○
You were bored by the program or activity	○	○	○	○	○	○	○	○	○	○	○
You felt pain when exercising	○	○	○	○	○	○	○	○	○	○	○
You had to exercise alone	○	○	○	○	○	○	○	○	○	○	○
You did not enjoy it	○	○	○	○	○	○	○	○	○	○	○
You were too busy with other activities	○	○	○	○	○	○	○	○	○	○	○
You felt tired	○	○	○	○	○	○	○	○	○	○	○
You felt stressed	○	○	○	○	○	○	○	○	○	○	○
You felt depressed	○	○	○	○	○	○	○	○	○	○	○

Please upload a screen shot of the exercise minutes recorded on your Apple Watch.

Instructions for retrieving Exercise Minutes:

1. Open the Health application on your iPhone. If you do not see this app on your phone, start typing “health” into the search bar.

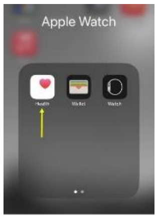

2. Select "Show All Health Data".

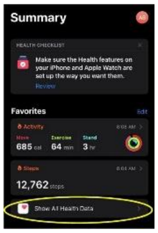

3. Select "Exercise Minutes".

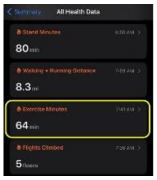

4. Select "Show All Data".

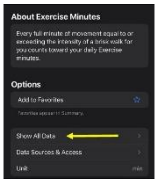

5. Be sure displayed on the screen are your exercise minutes over the past 8 days. Take a screenshot by pressing the volume up and power button at the same time. Upload this image from your photos on your iPhone.

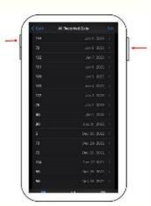

Choose file

Please provide your first and last name.

Please provide your age.

What is your affirmed gender?

- Male
- Female
- Non-binary/Third gender
- Prefer not to say

What is your current zip code?

Throughout the next 8 weeks, what type of environment will you reside in?

- o City dweller
- o Rural resident
- o Suburban resident

Are you currently pregnant or suspect pregnancy?

- o Yes
- o No

Has your physician told you that you should not presently participate in physical activity?

- o Yes
- o No

Mid-study survey - Completed at 4 weeks

This set of nine questions will be evaluating your confidence level regarding engagement in physical activity. Please select the response that best answers the question prompt right now.

0= Not confident
10= Very confident

	0 = Not confident	1	2	3	4	5	6	7	8	9	10 = Very confident
The weather was bothering you	○	○	○	○	○	○	○	○	○	○	○
You were bored by the program or activity	○	○	○	○	○	○	○	○	○	○	○
You felt pain when exercising	○	○	○	○	○	○	○	○	○	○	○
You had to exercise alone	○	○	○	○	○	○	○	○	○	○	○
You did not enjoy it	○	○	○	○	○	○	○	○	○	○	○
You were too busy with other activities	○	○	○	○	○	○	○	○	○	○	○
You felt tired	○	○	○	○	○	○	○	○	○	○	○
You felt stressed	○	○	○	○	○	○	○	○	○	○	○
You felt depressed	○	○	○	○	○	○	○	○	○	○	○

Please upload a screen shot of the exercise minutes recorded on your Apple Watch.

Instructions for retrieving Exercise Minutes:

1. Open the Health application on your iPhone. If you do not see this app on your phone, start typing "health" into the search bar.

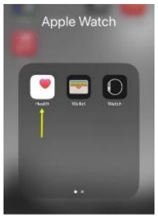

2. Select "Show All Health Data".

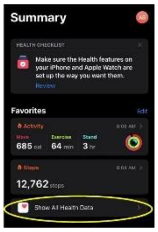

3. Select "Exercise Minutes".

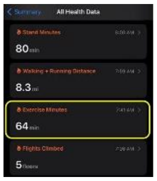

4. Select "Show All Data".

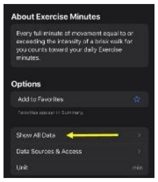

5. Be sure displayed on the screen are your exercise minutes over the past 8 days. Take a screenshot by pressing the volume up and power button at the same time. Upload this image from your photos on your iPhone.

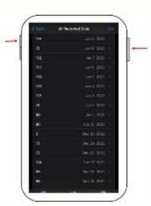

Choose file

Post-study survey for social feature users – Completed at 8 weeks

Thinking about your experience using the wearable activity tracker (Apple Watch) over the past eight weeks, please indicate the number between 1 and 10 that best represents your response.

1 = strongly disagree
5 = neither agree nor disagree
10 = strongly agree

	0	1	2	3	4	5	6	7	8	9	10
Did you find monitoring your activity rings on your Apple Watch to be a facilitator (help or improve) to your weekly physical activity?											
Did you use your Apple Watch to specifically monitor your daily exercise minutes (green ring)?											
Do you plan to continue to use your Apple Watch regularly to create and monitor personalized exercise goals?											
Do you find physical activity more pleasurable and/or satisfying since using the Apple Watch to monitor your activity habits during this study?											
Do you believe that from the start of this study to the end, you increased your total time spent per week doing moderate-to-vigorous physical activity (e.g., running, dancing, biking, water aerobics, brisk walking, doubles tennis, pick-up basketball, swimming laps)?											

The following questions are related to your use of the social features and sharing your fitness data with other Apple Watch users over the last 8-weeks.

1 = strongly disagree
5 = neither agree nor disagree
10 = strongly agree

	0	1	2	3	4	5	6	7	8	9	10
Did you find that sharing your activity data with digital connections on your Apple Watch was a greater facilitator (help or improve) to physical activity engagement than before you shared your activity data with others?											
Did you find the competition feature through activity sharing on the Apple Watch helpful at increasing your moderate-to-vigorous physical activity engagement?											
Did you find the competition feature through activity sharing on the Apple Watch helpful at improving your own belief in your ability to engage in physical activity?											
Did you find the competition feature through activity sharing on the Apple Watch helpful at improving your own belief in your ability to engage in physical activity?											
Did you find that being able to view others' daily activity on the Apple Watch was helpful to increase your moderate-to-vigorous physical activity engagement?											
Did you find that being able to view others' daily activity on the Apple Watch helpful to improve your own belief in your ability to engage in physical activity?											

Did you find the social support you received on your completed workouts and earned rewards provided by your digital connections on your Apple Watch was helpful to increase your moderate-to-vigorous physical activity engagement?	
Did you find the social support on your completed workouts and earned rewards provided by your digital connections on your Apple Watch helpful to improve your own belief in your ability to engage in physical activity?	
Were you satisfied with the level of social support you received from your digital connections on your Apple Watch about your physical activity engagement?	

Note the following terms for the next three questions:

- **Social Comparison:** evaluating and comparing of personal activity data to the activity data of those you are socially connected to on the device.
- **Competition:** active engagement in the device's competition feature.
- **Social Support:** active engagement in comments, feedback, and/or "likes" of social connections activity progress, workout completion, and/or earned rewards.

Which social feature did you engage with most frequently over the last 8-weeks on the device?

- O Social Comparison
- O Competition
- O Social Support

Which social behavior was most influential on increasing your weekly physical activity?

- O Social Comparison
- O Competition
- O Social Support

Which social behavior was most influential on your own belief in your ability to engage in physical activity (exercise self-efficacy)?

- O Social Comparison
- O Competition
- O Social Support

This set of nine questions will be evaluating your confidence level regarding engagement in physical activity. Please select the response that best answers the question prompt right now.

0= Not confident
10= Very confident

	0 = Not confident	1	2	3	4	5	6	7	8	9	10 = Very confident
The weather was bothering you	○	○	○	○	○	○	○	○	○	○	○
You were bored by the program or activity	○	○	○	○	○	○	○	○	○	○	○
You felt pain when exercising	○	○	○	○	○	○	○	○	○	○	○
You had to exercise alone	○	○	○	○	○	○	○	○	○	○	○
You did not enjoy it	○	○	○	○	○	○	○	○	○	○	○
You were too busy with other activities	○	○	○	○	○	○	○	○	○	○	○
You felt tired	○	○	○	○	○	○	○	○	○	○	○
You felt stressed	○	○	○	○	○	○	○	○	○	○	○
You felt depressed	○	○	○	○	○	○	○	○	○	○	○

Please upload a screen shot of the exercise minutes recorded on your Apple Watch.

Instructions for retrieving Exercise Minutes:

1. Open the Health application on your iPhone. If you do not see this app on your phone, start typing “health” into the search bar.

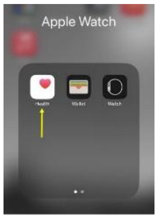

2. Select "Show All Health Data".

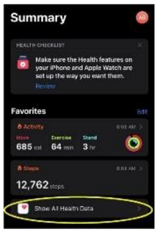

3. Select "Exercise Minutes".

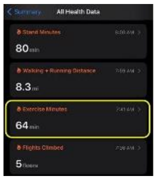

4. Select "Show All Data".

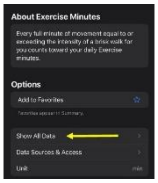

5. Be sure displayed on the screen are your exercise minutes over the past 8 days. Take a screenshot by pressing the volume up and power button at the same time. Upload this image from your photos on your iPhone.

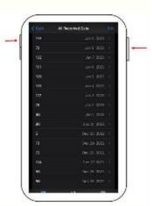

Choose file

Post-study survey for non-social feature users – Completed at 8 weeks

This set of nine questions will be evaluating your confidence level regarding engagement in physical activity. Please select the response that best answers the question prompt right now.

0= Not confident
10= Very confident

	0 = Not confident	1	2	3	4	5	6	7	8	9	10 = Very confident
The weather was bothering you	○	○	○	○	○	○	○	○	○	○	○
You were bored by the program or activity	○	○	○	○	○	○	○	○	○	○	○
You felt pain when exercising	○	○	○	○	○	○	○	○	○	○	○
You had to exercise alone	○	○	○	○	○	○	○	○	○	○	○
You did not enjoy it	○	○	○	○	○	○	○	○	○	○	○
You were too busy with other activities	○	○	○	○	○	○	○	○	○	○	○
You felt tired	○	○	○	○	○	○	○	○	○	○	○
You felt stressed	○	○	○	○	○	○	○	○	○	○	○
You felt depressed	○	○	○	○	○	○	○	○	○	○	○

Please upload a screen shot of the exercise minutes recorded on your Apple Watch.

Instructions for retrieving Exercise Minutes:

1. Open the Health application on your iPhone. If you do not see this app on your phone, start typing “health” into the search bar.

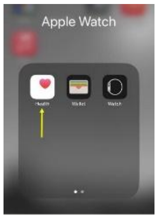

2. Select "Show All Health Data".

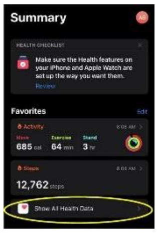

3. Select "Exercise Minutes".

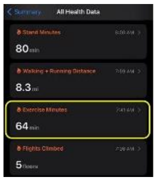

4. Select "Show All Data".

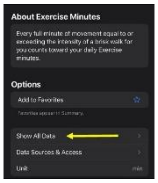

5. Be sure displayed on the screen are your exercise minutes over the past 8 days. Take a screenshot by pressing the volume up and power button at the same time. Upload this image from your photos on your iPhone.

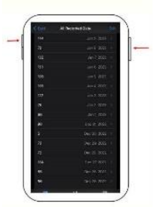

Choose file
