# Supplementary material for: Effect of Wearable Activity Tracker Social Behaviors on Physical Activity and Exercise Self-Efficacy: Real-World Pilot Study
Source: JMIR Form Res. 2026 May 5;10:e75133. doi: 10.2196/75133 (PMC13143198; doi:10.2196/75133)
Supplement: Multimedia Appendix 2 [file formative-v10-e75133-s002.pdf]

Supplementary File: Instructions for Sharing Fitness Activity & Engaging with Social Features on the Apple Wach

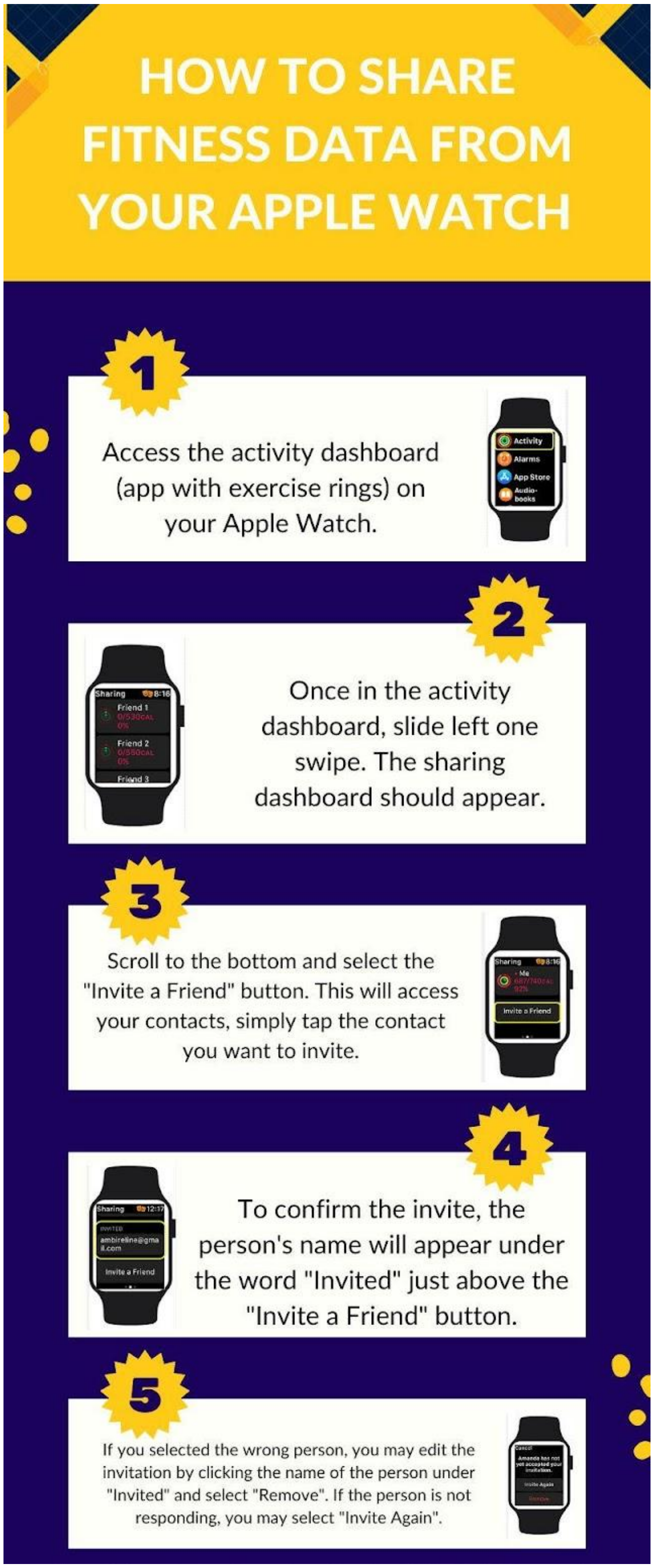

HOW TO SHARE FITNESS DATA FROM YOUR IPHONE

1

Access the fitness app (app with exercise rings) on your iphone.

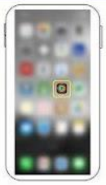

2

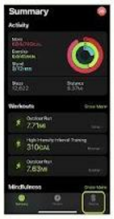

Select "Sharing" on the bottom right of the screen.

3

Select the icon in the top right corner with the green silhouette of a person and a + sign. Next, click the green + in the top right corner.

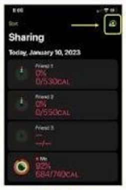

4

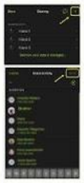

This should bring up your list of contacts. You may also manually add a phone number. Once you select someone (or more than one person), click send in the top right.

5

The person(s) you invited will appear on the screen until they have accepted the request. Once they have accepted, you will be able to view their fitness data on the sharing dashboard.

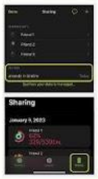

HOW TO ACCEPT AN INVITE TO SHARE FITNESS DATA

1

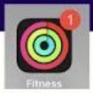

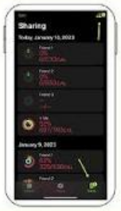

On your iPhone: You will receive a small notification symbol on the Fitness app. By opening the app, you will see a similar notification on the sharing symbol at both the bottom and top right of the screen. Select the top right icon.

2

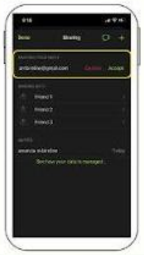

At the top of the screen, you will see "Accept" or "Decline" under "Awaiting Your Reply". By clicking "Accept" you agree to share your fitness data with the person who invited you.

3

On your Apple Watch: You will receive a notification of Activity Sharing asking if you would like to share your activity with the person who invited you.

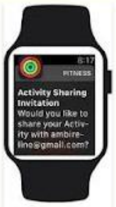

4

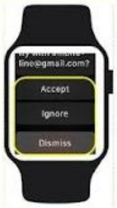

Simply scroll down and "Accept", "Ignore", or "Dismiss". By clicking "Accept" you agree to share your fitness data with the person who invited you.

USING SOCIAL FEATURES ON YOUR APPLE WATCH OR IPHONE

1

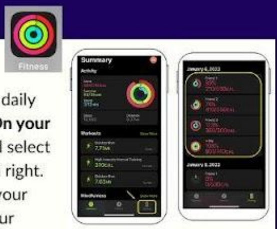

You can view your connections daily activity and compare your data! **On your iPhone**: Open the Fitness app and select the "Sharing" icon on the bottom right. Here you will be able to view your activity data compared to your connections.

2

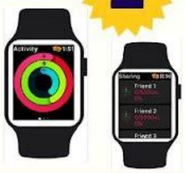

To compare your activity to your connections **on your Apple Watch**: Open the Activity app and swipe left one time. Here you will be able to view your activity data compared to your connections.

3

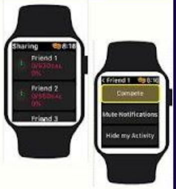

Challenge your connections to a competition! **On your Apple Watch**: Open the Activity app and swipe left one time. Select the connection you want to compete with. Scroll to the bottom and select "Compete". This invites them to a 7-day competition.

4

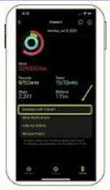

On your iPhone: open the Fitness app and select the "Sharing" icon on the bottom right. Select the connection you want to compete with by clicking their name. Select "Compete with *name of the person*" and this will invite them to a 7-day competition!

5

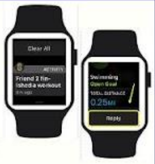

Support your friends when they complete a workout or fitness goal! You will receive a notification on your watch when your connection(s) complete a workout or earn an award. Click on the notification, click "Reply", and choose a pre-created comment and write your own customized message!
